# Classifying chemical sensor data using GPU-accelerated bio-mimetic neuronal networks based on the insect olfactory system

**DOI:** 10.1186/1471-2202-15-S1-P77

**Published:** 2014-07-21

**Authors:** Alan Diamond, Michael Schmuker, Amalia Z Berna, Stephen Trowell, Thomas Nowotny

**Affiliations:** 1 School of Engineering and Informatics, University of Sussex, Falmer Brighton, BN1 9QJ, UK; 2 Neuroinformatics & Theoretical Neuroscience, Inst. for Biology, Freie Universität Berlin, 14195 Berlin, Germany; 3 CSIRO Ecosystem Sciences and Food Futures Flagship, GPO Box 1700 Canberra, ACT 2601, Australia

## 

Chemosensing “e-nose” technology has great potential applications in everyday life, ranging from drug detection to food quality assessment and even the diagnosis of illness. However, odour detection and classification remains a highly challenging domain, characterized by high dimensionality, unknown organization of the vast “odourant space” of volatile chemicals and complex, turbulent odour plumes. To compound these difficulties, current sensor technology continues to exhibit distinct shortcomings in speed, sensitivity, selectivity, recovery, and drift avoidance.

In the research reported here we turn to a range of recent neuronal models [1-6] that were developed to describe the insect olfactory system, and have been shown to perform well across a range of classic, static classification tasks such as the MNIST handwritten digit set and the Sigma-Aldrich scent database. The insect olfactory system has been extensively studied and has been shown to be both fast and highly effective in complex natural conditions despite its limited size and complexity (when compared to the mammalian system) [2]. Insects such as moths, honey bees, locusts and fruit flies are capable of odour detection and classification tasks well beyond the abilities of current e-nose technology and machine learning algorithms [6].

We present results of applying an insect-inspired approach to the design of a learning spiking neural network that receives synchronized time series data from up to 12 metal-oxide based gas sensors, comprising an optimised [7] combination of classical doped tin oxide and novel zeolite-coated chromium titanium oxide sensors. We have collected sample data sets for classification tasks ranging from “easy” (single chemical identification presented under laboratory conditions) through to “hard” (identification of indicators of infectious diseases in breath samples taken from patients).

To address slow sensor response we consider a range of transient-based processing before applying self-organisation techniques to most effectively locate “virtual receptors” (VR) in sensor space. We look to address decorrelation (separation in feature space) and the supervised association of responses with rewards by using correlates of the insect antennal lobe (AL) and mushroom body (MB) structures whilst applying reward-based spike-timing dependent plasticity mechanisms.

Classification accuracy is compared with support vector machine (SVM) learning which we also look to match for speed through the use of GPU accelerated neural simulation [5] via the NVidia CUDA^TM^-based GeNN platform (http://sourceforge.net/projects/genn/).
